# Novel binders derived from an albumin-binding domain scaffold targeting human prostate secretory protein 94 (PSP94)

**DOI:** 10.1007/s13238-015-0194-9

**Published:** 2015-08-12

**Authors:** Lucie Marečková, Hana Petroková, Radim Osička, Milan Kuchař, Petr Malý

**Affiliations:** Institute of Biotechnology, Czech Academy of Sciences, v. v. i., Vídeňská 1083, 142 20 Prague, Czech Republic; Institute of Microbiology, Czech Academy of Sciences, v. v. i., Vídeňská 1083, 142 20 Prague, Czech Republic

**Dear Editor**,

Prostate secretory protein 94 (PSP94), also known as β-microseminoprotein, is a product of the *MSMB* gene and one of the most abundant proteins found in human seminal plasma (Anklesaria et al., 2013). This small cysteine-rich non-glycosylated protein is considered to be involved in regulation of many biological processes including male reproduction. It has been observed that PSP94 and its porcine homologue inhibit acrosomal reaction, sperm motility, and maintain the sperm environment (Anahi Franchi et al., 2008; Manaskova-Postlerova et al., 2011).

Despite of its possible role in reproduction, recently it has been proposed its association with development of prostate cancer (PC). It has been demonstrated that PSP94 suppresses tumor growth and reduces proliferation of some cancer cells by inducing apoptosis. This action is suggested to be regulated via binding to cell-surface receptors (Yang et al., 1998; Annabi et al., 2006). Moreover, the large genome-wide association studies showed that decreased expression of PSP94 caused by the rs10993994 single nucleotide polymorphism is associated with an increased risk of developing prostate cancer, suggesting a protective role of PSP94 in PC incidence (Lou et al., 2009; FitzGerald et al., 2013).

Currently, the clinically validated test for PC diagnosis relies on detection of serum level of prostate-specific antigen (PSA, human kallikrein-3) using monoclonal antibody-based ELISA kit but this examination, yet widely used, fails to predict early stages of PC development and, in addition, does not distinguish precisely between malign form of PC and benign prostate hyperplasia. Due to lack of this specificity many patients have to undergo unnecessary prostate tissue biopsy (Lazzeri et al., 2012). To overcome this drawback, a larger set of PC biomarkers has been suggested to improve early prediction of PC, to identify recurrent stages of malignancy after the prostatectomy and treatment, and to more precisely correlate serum-level oncomarkers with a histological Gleason scoring. Therefore, novel and more complex tools for improved PC diagnosis, including multifactorial biosensors or ELISA sets, are being required (Mhatre et al., 2014).

Many studies have suggested that PSP94 could be a useful biomarker for PC diagnosis and prognosis (Nam et al., 2006; Reeves et al., 2006; Velonas et al., 2013). The estimation of bound/free PSP94 has been suggested as an independent prognostic marker following radical prostatectomy that can predict time to recurrence of PC (Reeves et al., 2006). The measurement of PSP94 levels was able to identify patients with high-grade disease among a subset of patients in whom tests of PSA or free/total PSA were least informative (Nam et al., 2006; Mhatre et al., 2014).

Artificial binding proteins derived from small protein domain scaffolds represent a valuable non-immunoglobulin alternative for the construction of novel bio-sensing devices. Engineered small, stable, robust, and soluble proteins with a sufficient thermal and hydrodynamic stability and without disulphide bonds can be produced in a mass amount in bacteria and easily modified by gene-fusion approaches. In addition, they are amenable to rational improvement or *ab initio* design and suitable for high-throughput selection and diagnostic procedures (Gilbreth and Koide, 2012).

Recently we have demonstrated that a high-complex combinatorial library derived from three-helix bundle of albumin-binding domain (ABD) of streptococcal protein G can be used for development of sub-to-nanomolar affinity binders of human interferon gamma (Ahmad et al., 2012) or for novel IL-23 receptor antagonists with a promising anti-inflammatory potential (Kuchar et al., 2014). Therefore, we used this ABD scaffold-derived library to generate unique binders of human PSP94. In combination with five campaigns of ribosome display selection we generated a collection of 35 PSP94-binding clones called PAB binders, representing 29 unique sequence variants (Fig. 1A). These variants in the form of fusion proteins, carrying 46 amino acid residue-long ABD sequence linked to a helical 305 amino acid TolA spacer protein with an installed C-terminal AviTag consensus, were further characterized. PAB clones were tested for the production of bacterial proteins after transformation of *E. coli* host cells and protein production in cell lysates. After the verification of binding function in ELISA in combination with Western blot analysis, we selected most promising candidates for more detailed characterization. Purified recombinant proteins of these PAB variants confirmed binding to immobilized His-PSP94 bacterial protein in ELISA using detection with streptavidin-HRP conjugate (Fig. 1B). We also verified that these selected protein variants do not substantially bind to coated BSA protein (data not shown). To further confirm that these PAB clones bind to human PSP94 protein, we used another ELISA sandwich layout in which *in vivo* biotinylated PAB clones were immobilized to a coated streptavidin and binding of His-PSP94 was detected by anti-PSP94 antibody followed by a secondary IgG-HRP conjugate (results not shown).

**Figure 1:**
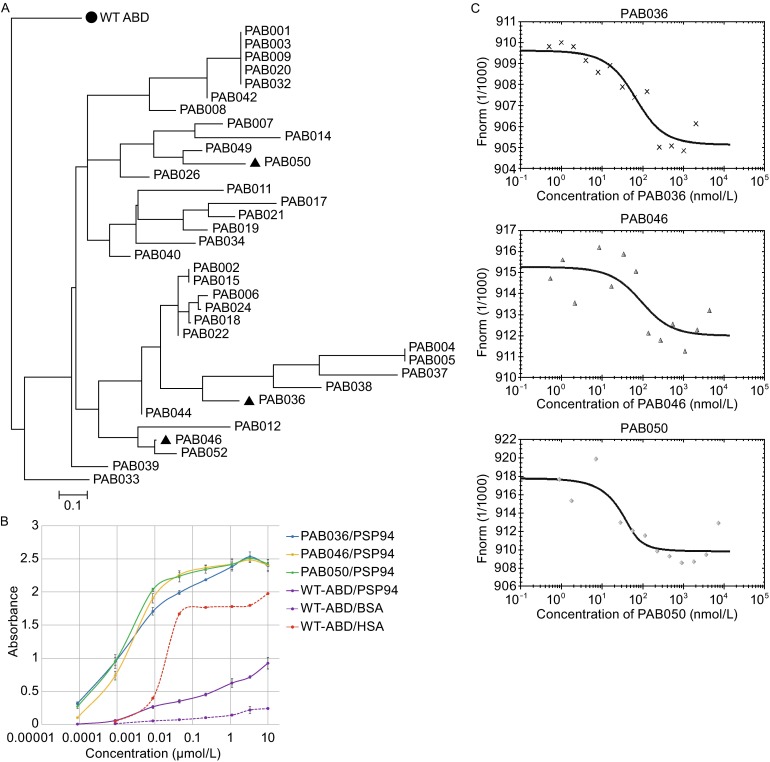
**Generation of PAB variants and their binding to recombinant HisTag-PSP94.** (A) Similarity tree of polypeptide sequences of the selected PAB binders. Analysis of a collection of 35 PAB binders obtained by ribosome display selection identified 29 unique sequence variants. For the analysis, randomized sequences between residues 20 and 46 were compared, as the N-terminal amino acid positions 1–19 were non-mutated. The sequence of the parental ABD wild-type domain (●) was used as a root of the tree. PAB variants selected for more detailed analysis are highlighted as triangles. (B) Binding of PAB variants to recombinant HisTag-PSP94 assessed by ELISA. Serially-diluted PAB variants in the form of biotinylated HisTag-PAB-TolA-AviTag fusion proteins were applied to a Polysorp microtiter plate coated with 10 μg/mL of recombinant HisTag-PSP94. WT-ABD indicates the parental wild-type ABD-TolA-AviTag protein as a non-mutated control with the natural affinity to HSA. The binding was detected by Streptavidin-HRP conjugate. The error bars represent the standard deviation from the three measurements. (C) The binding affinity of PAB variants to the fluorescently-labeled recombinant HisTag-PSP94 measured by microscale thermophoresis. Thermophoresis + T-jump data shown as binding curves were evaluated by a NanoTemper software and calculated *K*d values for PAB036, PAB046, and PAB50 variants were 40 ± 6 nmol/L, 49 ± 10 nmol/L, and 10 ± 3 nmol/L, respectively

To estimate the binding affinity of the particular PAB variants in solution, we used microscale thermophoresis (MST) with a fluorescently-labelled HisTag-PSP94 and measured interactions with PAB036, PAB046, and PAB050 variants. In the Fig. 1C, binding curves obtained for all selected PAB variants are shown, representing one of the performed experiments. Using a commercial software, *K*d constants measured for all three PAB variants were estimated to be 40 ± 6 nmol/L and 72 ± 25 for PAB036, 49 ± 10 nmol/L for PAB046, and 10 ± 3, 12 ± 3 and 21 ± 8 nmol/L (an average value 14 nmol/L) for PAB050.

To corroborate whether PAB binders are capable of binding to a native human target, we used LNCaP prostate cancer cell line formerly described to secrete and cell-surface express the PSP94 protein (Yang et al., 1998). To verify this expected positivity, we used flow cytometry to assay the binding of two monoclonal and one polyclonal anti-PSP94 antibodies. As demonstrated in the Fig. 2A, all three anti-PSP94 antibodies substantially bind to LNCaP cells while the presence of the only secondary Cy-5-conjugated IgGs remains to be negative. Statistical significance of the positive binding in comparison to the binding of the corresponding secondary antibody only was verified by ANOVA and is shown by asterisks. This result suggests that LNCaP cells capture a part of the secreted PSP94 by an autologous membrane receptor and can be, therefore, used for the investigation of binding of PAB clones to the membrane-bound PSP94. The possibility of the attachment of the PSP94 to cell surface via interactions with a membrane receptor has been already suggested (Annabi et al., 2006). Results of binding of all used anti-PSP94 antibodies to cell surface-bound PSP94 were further supported by intracellular binding of these antibodies to LNCaP cells with permeabilized membranes (results not shown). In the further experiment, *in vivo* biotinylated PAB variants in the form of PAB-TolA-AviTag fusion proteins were tested by flow cytometry for the ability to bind to PSP94-expressing LNCaP cells. The ABD-wild-type protein corresponding to the parental non-randomized ABD scaffold was used as a control to exclude the possibility that non-randomized residues of the ABD bundle could mediate high-affinity interactions with prostate cancer cells. In addition, presence of TolA fusion moiety, identical between PAB variants and the ABD wild-type control, should exclude that ABD-unrelated TolA sequences mediate or substantially contribute to cell-surface binding. As demonstrated in the Fig. 2B, several PAB variants bind to LNCaP cells, as detected by streptavidin-PE conjugate. Statistical significance of binding for each PAB variant compared to the binding of parental wild-type ABD-TolA control is provided by ANOVA and shown by asterisks. We also investigated binding of PAB clones to permeabilized LNCaP cells and results are in correlation with intact cells staining (data not shown). Interestingly, PAB036 variant, binding well to the bacterial PSP94 in ELISA, and substantially also to the intracellular LNCaP cell product (not shown), exhibited weaker cell-membrane binding compared to the other PAB variants. It is possible that this clone recognizes a different binding surface of the PSP94 in comparison to the other binders and that this membrane PSP94, bound in an oriented way to its cognate cell-surface receptor, is sterically hindered and, thus, partially unapproachable for a full PAB036 recognition. This is supported by a strong binding in the case of a free accessible PSP94 (data not shown). Based on the result of this binding assay, we selected PAB036, PAB046, and PAB050 variants as the most promising candidates for further characterization. The amino acid sequences between residues 20 and 46 of these three variants are as follows: PAB036: **W**YKN**G**IN**P**A**HR**V**RW**VK**GR**ID**A**ILA**R**LP; PAB046: **R**YKN**A**IN**R**A**PA**V**WW**VK**RL**ID**A**ILA**A**LP; PAB050: **L**YKN**H**IN**T**A**WR**V**AA**VK**RA**ID**L**ILA**S**LP, all presented with the indicated positions of the 11 randomized residues. Parental non-randomized WT-ABD full-length sequence (46 amino acids of the full ABD scaffold) is LAEAKVLANRELDKYGVSD**Y**YKN**L**IN**N**A**KT**V**EG**VK**AL**ID**E**ILA**A**LP with the marked mutable positions.

**Figure 2:**
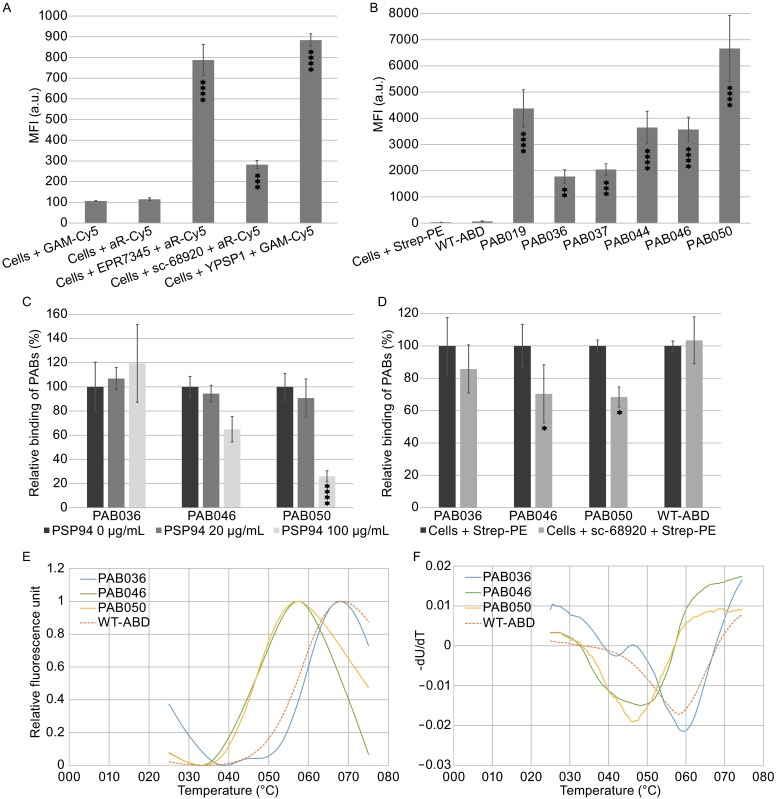
**Binding of PAB variants to prostate cancer LNCaP cells analyzed by flow cytometry and tests of their thermal stability.** (A) Detection of PSP94 on cell-surface of prostate cancer LNCaP cells using anti-PSP94 antibodies: monoclonal EPR7345, polyclonal sc-68920, and monoclonal YPSP-1. (B) Binding of PAB-TolA-Avitag variants to LNCaP cells. Soluble PAB proteins purified as *in vivo* biotinylated HisTag-PAB-TolA-AviTag fusion products were added to cells and the binding was detected by streptavidin-PE conjugate. WT-ABD indicates parental non-mutated ABD wild-type as a control. (C) Competition of recombinant HisTag-PSP94 with the selected PAB variants for binding to LNCaP cells. The graphical representation of binding of *in vivo* biotinylated PAB-TolA-Avitag variants to LNCaP cells in the presence of increased concentrations of recombinant HisTag-PSP94 is shown, as detected by streptavidin-PE conjugate. The fluorescent intensities of bound PAB clones to LNCaP cells in the absence of recombinant PSP94 were taken as 100% and the averaged values of the three experiments are shown with standard deviations. (D) Competition of rabbit polyclonal antibody sc-68920 with PAB variants for binding to membrane-bound PSP94 on LNCaP cells. Cells were incubated with (80 µg/mL) or without (control) sc-68920 antibody for 15 min on ice, then PAB binders at concentration 10 µg/mL were added and left to incubate for 30 min. Binding of PAB variants was detected by streptavidin-PE conjugate. In all flow cytometry binding tests (A–D), results are expressed as the arithmetic mean ± standard deviation of the mean. Statistical analysis was done using one-way ANOVA followed by Dunnett’s post-test, comparing all the samples with the control. GraphPad Prism 6.0 (GraphPad Software) was used to perform statistical analysis. Significant differences are indicated by asterisks (*, *P* < 0.05; **, *P* < 0.01; ***, *P* < 0.001; ****, *P* < 0.0001). (E) Thermal melting fluorescence curves of PAB binders and parental non-mutated ABD wild-type (WT-ABD) control. (F) First derivative of fluorescence versus temperature of curves shown in the panel (E). The melting point is given as the lowest point of the curve. All measurements were done in duplicate (PAB046) or in triplicates (PAB036, PAB050, WT-ABD) and averaged

To verify whether recombinant bacterial human PSP94 competes with cell-bound PSP94, we performed cell-surface competition binding assay in which PAB036, PAB046, and PAB050 variants were mixed with an increasing concentration of the recombinant PSP94 protein and were left bound to LNCaP cells for 30 min. The results of repeated experiments shown in the Fig. 2C indicate that increasing concentrations of the PSP94 protein inhibited binding of the PAB046 and PAB050 variants to the cells. While the concentration 20 µg/mL decreased binding of both these PAB proteins only by 5%–10%, concentration of 100 µg/mL inhibited this binding substantially by 35% for PAB046 and by 74% for PAB050. Contrary that, PAB036 variant did not exhibit any inhibitory effect. Statistical evaluation by ANOVA was performed and indicated by asterisks.

As an important proof of specificity of PAB binders, we performed cell-surface competition binding assay using LNCaP cells. We first tested whether monoclonal antibody YPSP-1 inhibits binding of PAB variants to LNCaP cells by flow cytometry. Our data demonstrated that this monoclonal antibody did not compete with PAB036, PAB046, and PAB050 variants for binding to LNCaP cells (data not shown), suggesting that an interacting epitope differs from those recognized by the particular PAB binders. We also tested the ability of polyclonal antibody sc-68920 to inhibit binding of PAB variants to the same cells and found that this antibody is able to suppress the PAB binding. We used it, therefore, in cell-surface competition binding assay to demonstrate the specificity of used PAB binders for cell-bound PSP94 recognition on LNCaP cells. As shown in Fig. 2D, a dose of 4 micrograms of sc-68920 suppressed the binding of PAB046 and PAB050 by 30% compared to the control non-inhibited PAB variants (100%) and this decrease is statistically significant as verified by ANOVA (indicated by asterisks). On the other hand, inhibition of PAB036 binding was only about 15%. ABD-WT-TolA protein was used as a non-inhibiting ABD control in this flow cytometry test. To investigate what is the impact of ABD scaffold randomization on PAB binder’s stability, we measured thermal stability of PAB036, PAB046, and PAB050 variants using thermal shift assay (TSA). Temperature melting points (Tm) measured in 300 mmol/L NaCl, 50 mmol/L Tris buffer, pH 8, are 59.6°C for PAB036, 48.5°C for PAB046, and 46.0°C for PAB050 (Fig. 2E and 2F). Tm value for parental non-randomized wild-type ABD is 58.0°C. These data indicate that particular mutations in each of variants can strongly affect the protein stability. While in the case of PAB036 variant the amino acid alterations slightly improved the original scaffold stability, thermal stability of PAB046 as well as PAB050 was significantly decreased.

Collectively, we present the generation and characterization of unique protein binders of human prostate cancer oncomarker that can be useful as alternatives to monoclonal antibodies for detection of MSMB in studies of fertilization and, with possible modifications using gene-fusion or affinity maturation approaches, they could serve as novel capture proteins for improved prostate cancer diagnostics.

## Electronic supplementary material

Supplementary material 1 (PDF 175 kb)
