# Supplementary material for: Novel binders derived from an albumin-binding domain scaffold targeting human prostate secretory protein 94 (PSP94)
Source: Protein Cell. 2015 Aug 12;6(10):774–9. doi: 10.1007/s13238-015-0194-9 (PMC4598320; doi:10.1007/s13238-015-0194-9)
Supplement: Supplementary file 1 — Supplementary material 1 (PDF 175 kb) [file 13238_2015_194_MOESM1_ESM.pdf]

A SUPPLEMENTARY FILE

MATERIALS AND METHODS

Cell lines and growth conditions

Cells of androgen-sensitive human prostate adenocarcinoma cell line LNCaP (clone FGC, ATCC number: CRL-1740) were grown in RPMI-1640 medium (Sigma-Aldrich, St. Louis, MO) supplemented with 10% fetal calf serum (FCS) (GIBCO, Grand Island, N.Y.) and antibiotic antimycotic solution (ATB) (Sigma-Aldrich, St. Louis, MO) in 5% CO_2 at 37 °C.

Antibodies and detection agents

Mouse monoclonal antibody to prostate secretory protein (PSP) [YPSP-1] (ab19070), rabbit monoclonal antibody to PSP [EPR7345] (ab128897) (Abcam®, Cambridge, UK) and rabbit polyclonal antibody PSP94 (H-114): sc-68920 (Santa Cruz Biotechnology Inc., Dallas, USA) were used. As secondary reagents, Thermo Scientific™ Pierce™ Streptavidin HRP Conjugate, High Sensitivity *(*Thermo Fisher Scientific Inc.*)*, Cy5-conjugated goat anti-mouse IgG F(ab′)$_2$ fragment (aM-Cy5) (Jackson ImmunoResearch Laboratories, West Grove, PA), Cy5-conjugated goat anti-rabbit IgG F(ab′)$_2$ (aR-Cy5) (Jackson ImmunoResearch Laboratories, West Grove, PA) or Streptavidin-phycoerythrin (eBioscience, San Diego, CA) were used.

Production of recombinant forms of PSP94

pDNR-LIB plasmid vector carrying cDNA for expression of 114 amino acid residue-long mature PSP94 was purchased from Geneservice Ltd. (Cambridge, UK) (GenBank accession no. BC005257.1). For bacterial production of a PSP94 variant carrying polyhistidinyl tag at the N-terminus (HisTag-PSP94), the DNA sequence lacking a leader peptide secretory consensus

was amplified by PCR using a forward primer ACTTCATATGTCATGCTATTTCATACCTAATG, containing ATG start codon and *Nde*I cloning site, and a reverse primer GCGGATCCTTAGATTATCCATTCACTGACA, containing the stop codon and *Bam*HI cloning site. The resulting PCR product was inserted into the pET-28b vector (Novagene, Germany) using *Nde*I/*Bam*HI cloning sites and introduced into *E. coli* DH5α host cells (New England Biolabs, Ipswich, MA) or, for protein production, into ArcticExpress (DE3) *E. coli* cells (Agilent Technologies, US). A cytosolic extract was then purified by affinity chromatography using Ni-NTA agarose (Qiagen, Germany) and elution fractions were further purified by gel permeation chromatography (Äkta^{TM} Purifier; column Superdex 75, 16/60). Eluted fractions in 50 mM Tris, 150mM NaCl, pH 8.0 (T50N150) were analyzed by SDS-PAGE and Western Blot.

Selection of PSP94-binding variants by ribosome display

Combinatorial DNA library was generated as described previously (Ahmad et al., 2012; Kuchar et al., 2014). The five rounds of ribosome display selection were carried out according to (Kuchar et al., 2014) using an improved forward primer EWT5-ABDforN1 (TTCCTCCATGGGTATGAGAGGATCGCAT CACCATCACCATCACCTGGCGGAAGCTAAAGTCTTAGCTAAC) replacing the former EWT5-ABDforN. After the final round of the selection, forward primer EWT5-ABDforN2 (TTCCTCCATGGGCAGCAGCCATCACCATCACCATCACCTGGCGGAAGCTAAAGT CTTAGCTAAC) instead of the former EWT5-ABDforN was used in PCR to produce full-length tolA fusion sequence. Resulting 6xHis-PAB-TolA-AviTag fusion variants were produced as *in vivo* biotinylated proteins in *E. coli* BL21 (DE3) BirA strain and purified from cell lysates by affinity chromatography according to NiNTA Spin Column protocol (Qiagen, Germany).

Sequence analysis and clustering of selected PAB variants

DNA of clones expressing PAB variants was verified by sequencing. Amino acid sequence alignment of all selected clones was used to generate a similarity tree using Molecular Evolutionary Genetics Analysis version 5 (MEGA5) integrated tool (http://www.megasoftware.net).

Test of binding of PAB variants to PSP94 by ELISA

Polysorp microplate (NUNC, Denmark) was coated with 10 µg/mL of the recombinant PSP94 diluted in coating buffer (100 mM bicarbonate/carbonate solution, pH 9.6) at 4 °C overnight. The plate was washed with PBST buffer (PBS buffer with 0.05% Tween-20) and blocked with 1% BSA in PBST buffer for 2 hours at room temperature. After washing, serially-diluted *in vivo* biotinylated PAB clones or ABD-WT control in PBST/1% BSA were added. Bound PAB variants were detected by streptavidin-HRP conjugate (diluted 1:5000). The plate wells were washed repeatedly and stained in 0.1M citrate buffer, pH 5.0 containing 0.5 mg/mL o-phenylenediamine (OPD substrate, Sigma-Aldrich, St. Luis, MO) and 0.03% H_2O_2 for 5 min. The colorimetric reaction was stopped with 100 µL 2M H_2SO_4 and absorbance at 492 nm was determined.

Detection of cell-bound PSP94 using flow cytometry

Scraped LNCaP cells were washed with HBSS Complete buffer (10 mM HEPES, pH 7.4, 140 mM NaCl, 5 mM KCl, 1% (v/v) glucose, 2 mM $CaCl_2$, 2 mM $MgCl_2$, 1% (v/v) FCS), added to 96-well plate (NUNC, Roskilde, Denmark) to final number of $5x10^5$ LNCaP cells per well, centrifuged, and the buffer was removed. The cells were incubated separately with 50 µL of diluted primary antibodies against PSP94 (EPR7345, sc-68920, YPSP-1) (diluted 1:100 with

HBSS Complete buffer) for 30 min at 4 °C. The cells were washed with HBSS Complete buffer and incubated with 100 µL of diluted fluorescent labeled secondary antibodies (aR-Cy5, aM-Cy5) (diluted 1:500) for 30 min at 4 °C. After washing, the cells were re-suspended in 100 µL of HBSS Complete buffer and analyzed by flow cytometry using a FACS LSR II instrument (BD Biosciences, San Jose, CA) in the presence of 1 µg/mL of Hoechst 33258. Data were analyzed using the FlowJo software (Tree Star, Ashland, OR). Appropriate gatings were used to exclude cell aggregates and dead cells and binding data were expressed as mean fluorescence intensity (MFI) values.

Binding of PAB variants to cell-bound PSP94

Scraped LNCaP cells were washed with HBSS Complete buffer, added to 96-well plate to final number of $7.5x10^5$ LNCaP cells per well, centrifuged, and the buffer was removed. The cells were incubated with 100 µL of PAB clones or ABD-WT control diluted to a final concentration of 10 µg/mL in HBSS Complete buffer for 30 min at 4 °C. After washing, 50 µL of diluted Strep-PE (diluted 1:400 with HBSS Complete buffer) was added, and the cells were incubated for 30 min at 4 °C. Washed cells were resuspended in 100 µL of HBSS and analyzed by FACS. Relative binding of PAB variants was calculated as MFI of cells treated with PAB and Strep-PE/MFI of cells treated with Strep-PE.

Cell-surface competition binding assay

HisTag-PSP94 diluted to a final concentration of 20 µg/mL or 100 µg/mL in HBSS Complete buffer was mixed with PAB clones or WT-ABD diluted to a final concentration of 5 µg/mL (in 50 µL), and pre-incubated for 15 min on ice. Scraped LNCaP cells were washed with HBSS Complete buffer, added to 96-well plate to final number of $2.5x10^5$ LNCaP cells per well, centrifuged, and the buffer was removed. The cells were incubated with prearranged mixture

of PAB variants with HisTag-PSP94 for 30 min on ice. After washing, 50 µL of diluted Strep-PE (diluted 1:400) was added, and the cells were incubated for 30 min at 4 °C. Washed cells were re-suspended in 100 µL of HBSS and analyzed by FACS as described above. For statistical comparison, the Student's t-test was used and for all experiments p-value of 0.05 was considered significant.

Test of binding affinity by microscale thermophoresis (MST)

For fluorescent labeling of the PSP94, purified HisTag-PSP94 was dialyzed into PBS buffer pH 7.4 and labeled following the protocol (L001 ™ Monolith NT.115 Protein Labeling Kit RED-NHS, NanoTemper Technologies GmbH, Germany). The stock solution of labeled PSP94 was diluted in T50N150 buffer at final concentration 50 nM. Decreasing concentrations of non-labeled purified PAB variants were titrated against labeled HisTag-PSP94 and centrifuged for 10 min at 15,000 g to remove potential aggregates. The supernatant was soaked into standard capillaries (NanoTemper Technologies GmbH). Measurements were carried out at 25 °C in T50N150 buffer containing 0.025% Tween20 on the Monolith NT.115 instrument (NanoTemper Technologies GmbH) using 10% LED power and 20% and 40% MST power. In some cases, several independent measurements were performed. Data analysis was completed with NanoTemper analysis software.

Fluorescence-based thermal-shift assay (TSA)

Protein samples (0.2 mg/mL) in T50N300 buffer and T50N300 buffer with 500 mM imidazole, and 5× SyproOrange dye (Sigma-Aldrich, St. Luis, MO) were added into 25 µL total volume. The solutions were incubated in a thermal gradient and evaluated as described previously (Kuchar et al., 2014).
